# The Bilateral Precuneus as a Potential Neuroimaging Biomarker for Right Temporal Lobe Epilepsy: A Support Vector Machine Analysis

**DOI:** 10.3389/fpsyt.2022.923583

**Published:** 2022-06-15

**Authors:** Chunyan Huang, Yang Zhou, Yi Zhong, Xi Wang, Yunhua Zhang

**Affiliations:** ^1^Wuhan Third Hospital, Tongren Hospital of Wuhan University, Wuhan, China; ^2^Wuhan Mental Health Center, Wuhan, China; ^3^Wuhan Hospital for Psychotherapy, Wuhan, China; ^4^NHC Key Laboratory of Mental Health (Peking University), Peking University Institute of Mental Health, Peking University Sixth Hospital, Beijing, China; ^5^Department of Sleep and Psychosomatic Medicine Center, Taihe Hospital, Affiliated Hospital of Hubei University of Medicine, Shiyan, China; ^6^Hubei Provincial Hospital of Traditional Chinese Medicine, Wuhan, China; ^7^Clinical Medical College of Hubei University of Chinese Medicine, Wuhan, China; ^8^Hubei Province Academy of Traditional Chinese Medicine, Wuhan, China

**Keywords:** right temporal lobe epilepsy, resting-state functional magnetic resonance, default mode network, network homogeneity, biomarker

## Abstract

**Background and Objective:**

While evidence has demonstrated that the default-mode network (DMN) plays a key role in the broad-scale cognitive problems that occur in right temporal lobe epilepsy (rTLE), little is known about alterations in the network homogeneity (NH) of the DMN in TLE. In this study, we used the NH method to investigate the NH of the DMN in TLE at rest, and an support vector machine (SVM) method for the diagnosis of rTLE.

**Methods:**

A total of 43 rTLE cases and 42 healthy controls (HCs) underwent resting-state functional magnetic resonance imaging (rs-fMRI). Imaging data were analyzed with the NH and SVM methods.

**Results:**

rTLE patients have a decreased NH in the right inferior temporal gyrus (ITG) and left middle temporal gyrus (MTG), but increased NH in the bilateral precuneus (PCu) and right inferior parietal lobe (IPL), compared with HCs. We found that rTLE had a longer performance reaction time (RT). No significant correlation was found between abnormal NH values and clinical variables of the patients. The SVM results showed that increased NH in the bilateral PCu as a diagnostic biomarker distinguished rTLE from HCs with an accuracy of 74.12% (63/85), a sensitivity 72.01% (31/43), and a specificity 72.81% (31/42).

**Conclusion:**

These findings suggest that abnormal NH of the DMN exists in rTLE, and highlights the significance of the DMN in the pathophysiology of cognitive problems occurring in rTLE, and the bilateral PCu as a neuroimaging diagnostic biomarker for rTLE.

## Introduction

Temporal lobe epilepsy (TLE), the most common form of adult epilepsy, is a common nervous system disease (1, 2). It is characterized by complex partial seizures, and secondary generalizations resulting from abnormal electrical activity in the temporal lobe, presenting as epileptic foci (3, 4). The recurring seizures in most people with TLE, result in cognitive dysfunction in areas such as learning, language, memory, emotion, perception, attention, consciousness, and behavior, which has a serious impact on their cognitive abilities and their lives (5–7). Existing studies have shown that the pathogenesis of epilepsy can be further understood through the study of the brain network properties, and interactions between different brain regions. However, the exact mechanism of the onset of this disorder is still not clear.

Advances in neuroimaging techniques have enabled increasingly detailed observation of alterations in the brain that are involved in the pathophysiology of TLE. Such observations have shown that topological patterns of brain structural networks were aberrant in patients with TLE (8). Abnormalities of the uncinate fasciculus correlate with executive dysfunction in patients with left TLE (9). Resting-state functional magnetic resonance imaging (fMRI), has a potential to detect abnormal neural activity, and is therefore extensively used in neuroscience. Up to the present, there are a large number of studies using fMRI to study TLE, investigating diverse abnormalities in different brain regions (10–15). However, according to the findings of these studies, the pathophysiology of TLE is still unclear.

In recent, the growing body of functional neuroimaging, at-rest data has opened up new avenues for surveys of the previously neglected field of intrinsic network organization. TLE is increasingly thought to be a disorder involving abnormal epileptogenic networks, rather than a single focal epileptogenic source (16–18). Accumulating evidence has shown that TLE exists in several networks disturbances, including alertness network (19), attention network (20) and default mode network (DMN) (13, 21, 22). However, there are only a few reports concerning the DMN and its function in patients with TLE.

Interestingly, the DMN has received increasing attention because it plays important roles in many medical or neurological illnesses. This network is characterized by showing higher activity at rest, deactivating during task-related cognitive processes (23, 24). Recently, the DMN was thought to include several special brain regions, such as medial prefrontal cortex (MPFC), lateral posterior cortices, posterior cingulate cortex / precuneus (PCC / PCu) (25), lateral temporal gyrus (26), cerebellar Crus 1 and Crus 2 (27). Researchers have demonstrated that the DMN is closely correlated with episodic memory processing, negative ruminations, complex self-referential stimuli (28) and in some special mind-states, such as anesthesia and sleep (29). Furthermore, the DMN is associated with cognitive functioning, especially executive function (30).

In addition, increasing evidence has shown a connectivity of abnormal resting state within the DMN in patients with epilepsy, but the results are mixed. For instance, many findings showed that there are increased PCC, and decreased medial prefrontal cortex (MPFC) functional connectivities in TLE (31, 32). However, other studies found decreased DMN connectivities in PCC, anterior frontal, and parietal regions (33, 34). Moreover, antiepileptic drugs and duration of illness could also contribute to the abnormality of DMN (35). These findings consistently show that the DMN plays a crucial role in TLE. However, the homogeneity of this network has not been fully explored.

Recently, the SVM is widely used in neuropsychiatric diseases due to its scientificity and effectivity (10, 11, 15, 36–38). An optimal separating hyperplane of the high-dimensional space can be confirmed by the SVM. In the fMRI analysis, a discrimination map can be generated by superimposing the SVM weights back to the original brain space, and the most significant weights can be visually traced back to the most discriminative parts of the brain. The SVM method has great potential to provide clinically useful criteria for decision-making from such high-dimensional neuroimaging data. In this study, we investigated NH of DMN in rTLE patients, and hypothesized NH values in altered brain regions could be used as potential neuroimaging biomarkers to diagnose rTLE through the SVM method. In this work, we used a method called network homogeneity (NH) (39) to analyze resting state data in TLE. This informative approach studies a given network without specifying the location of network abnormalities. It assesses the correlation of a voxel with all other voxels within a specific network of interest. Homogeneity is defined to be the average correlation of the time series of any given voxel with the time series of all other voxels within the network. The NH method has been used for depression, somatization, attention-deficit/hyperactivity disorder, schizophrenia and their unaffected siblings (39–46). Epilepsy encompasses different epileptic types with differing discharge places, which illustrates the differences in structure and functional impairment are possible (31). Even in identical brain regions, the left and right sides show differences (47). Thus, studies on unilateral TLE may have the advantage when assessing brain function, because it lessens the confounding effects of differences in discharging places. Using the NH method, we studied the NH of the DMN in people with rTLE, and examined the characteristics of the DMN and possible mechanism that causes rTLE in patients with rTLE. Furthermore, we try to find a potential biomarker to diagnosis rTLE from healthy controls (HCs).

## Materials and Methods

### Subjects

A total of 43 patients with rTLE were recruited from the Epilepsy Clinic at the Department of Neurology, Sleep and Psychosomatic Medicine Center, Taihe Hospital, Hubei University of Medicine. The diagnosis of rTLE was made according to the diagnostic criteria of the International League Against Epilepsy (48). Patients with epilepsy who met any two of the following symptoms were classified as patients with rTLE: (1) the clinical onset of symptoms suggested the location of epileptogenic focus in the temporal lobe; (2) interictal electroencephalographic (EEG) traces illustrated lesions in the right temporal lobe; and (3) an MRI showed sclerosis or atrophy in the right temporal lobe. Exclusion criteria were as follows: left-handed; pregnant or breastfeeding, history of tobacco, alcoholic, drug abuse, and history of serious medical diseases; mental disorders or other neurological illnesses; a score <24 in a mini-mental state examination (MMSE), and contraindications for MRI.

A total of 42 age-, gender-, and years of education- matched healthy controls were recruited from the community. Exclusion criteria for the healthy controls were: (1) history of brain operations; (2) history of severe neuropsychiatric diseases; (3) serious medical illness; and (4) pregnant or breastfeeding; history of tobacco, alcoholic, drug abuse. Participants who had any contraindications for MRI were excluded. Patients and healthy controls were subjected to an MMSE to evaluate cognitive function. The reaction time (RT) measurements obtained from the Attentional Network Test (49) were used to assess executive function. All participants provided a written informed consent before entering the study. The ethics committee of the Taihe Hospital, Hubei University of Medicine approved the study.

### Scan Acquisition

Scanning was conducted using an Achieva 3T MRI scanner (Philips, Netherlands). Participants were asked to lie down with their eyes closed and to remain awake. We used a prototype quadrature birdcage head coil fitted with foam padding to minimize head movement. The scanning parameters were as follows: (1) structural scan (T1-weighted): spin-echo sequence, repetition time (TR) = 20 ms, echo - time (TE) = 3.5 ms, slice thickness = 1 mm, and field of view (FOV) = 24^*^24 cm, scan time about 7 min. (2) rs - fMRI scan: gradient echo - echo planar imaging sequence (echo - planar imaging T2^*^ weighted), TR/TE = 2,000/30 ms, slice thickness = 5 mm, pitch = 1 mm, FOV = 220 × 220 mm^2^, and flip angle = 90°, scan time was about 9 min.

### Data Preprocessing

DPARSF software (50) was used in MATLAB for preprocessing rs-fMRI imaging data. First, the first 5 time points were discarded. Then, slice-time and head-motion correction were performed. At this point, participants who had more than 2 mm of maximal displacement on the x, y, or z axis, and more than 2°of maximal rotation were excluded. Subsequently, normalization and resampling were performed to generate the dimensions of 3 × 3 × 3 mm. During the process of functional image normalization, head motion parameters, white matter signal and cerebrospinal fluid signal were used as removal covariates, and a voxel size of 3 x 3 x 3 mm was used as a functional covariate. After that, an 8 mm, full-width at half-maximum Gaussian kernel was used to smooth the acquired images. Temporal bandpass filtering (0.01–0.08 Hz) and linear detrending were used to reduce the influence of low-frequency drifts, and physiological high-frequency noise. During preprocessing, the signal from a region centered in the white matter, six head motion parameters obtained by rigid body correction, and signal from a ventricular region of interest were removed. However, the global signal was preserved, given that removal may introduce artifacts into the data and distort resting-state connectivity patterns, and the regression of the global signal may significantly distort results when studying clinical populations (51, 52).

### DMN Identification

The group independent component analysis (ICA) method was used to pick out DMN components according to the templates provided by GIFT (53). Briefly, the ICA analysis included three main steps using the GIFT toolbox (52): data reduction; independent component separation; and back reconstruction. The generated DMN was used as a mask for further NH analyses.

### NH Analysis

NH analysis was performed using an in-house script in Matlab. For each subject, the correlation coefficient of each voxel was computed against all other voxels within the DMN mask. Then, the mean correlation coefficient was averaged and subsequently changed into a z-value by using a z-transformation. The resultant values generated the NH maps. Finally, the NH maps were z-transformed for group comparison.

### Statistical Analyses

Demographic information, including age, gender, years of educational, and imaging data were compared between the rTLE and the HCs. Chi-square test and the two-sample *t*-test were, respectively used to compare the categorical data and continuous variables. The NH maps of patients and HCs were analyzed with a two-sample *t*-test via voxel-wise cross-subject statistics within the DMN mask. The significance level was set to be *p* < 0.01, and corrected for multiple comparisons using Gaussian Random Field (GRF) theory (GRF corrected, voxel significance: *p* < 0.001, cluster significance: *p* < 0.01).

### Classification Analyses

LIBSVM (a Library for Support Vector Machines) software package was applied to examine whether abnormal NH in the DMN could be used as potential biomarkers for diagnosis of rTLE.

## Results

### Demographics and Clinical Characteristics of the Subjects

No patients or controls were excluded due to excessive head movement. No significant differences were found between the two groups in terms of gender, age, years of education and MMSE. The rTLE group had longer RTs, but no significant differences in RT were found between the rTLE group and the HCs. The demographic data for the recruited subjects are given in Table 1.

**Table 1:** Characteristics of the participants.

**Demographic data**	**Patients (*n* = 43)**	**NC (*n* = 42)**	**T (orx^**2**^)**	***P*** **-value**
Gender (male/female)	43 (23/20)	42(22/20)	0.12	0.29^a^
Age (years)	27.91 ± 6.48	26.96 ± 5.31	0.74	0.46^b^
Years of education (years)	13.01 ± 2.67	13.67 ± 1.88	−1.33	0.19^b^
Illness duration (years)	8.49 ± 7.1	67.81 ± 48.02	2.13	0.04^b^
ECRT	91.60 ± 54.85			

a *The p-value for gender distribution was obtained by chi-square test*.

b *The p-value were obtained by two sample t-tests*.

*NC, normal control; ECRT, executive control reaction time*.

### The DMN Maps Determined by Group ICA

The DMN mask was constructed from the control group using the ICA method. The DMN included the following brain regions: bilateral MPFC; PCC/PCu; ventral anterior cingulate cortex; lateral temporal cortex; medial, lateral, inferior parietal lobes; and cerebellum Crus 1 and Crus 2. The generated DMN mask was used in the subsequent NH analysis.

### NH: Group Differences in the DMN

**Figure 1:**
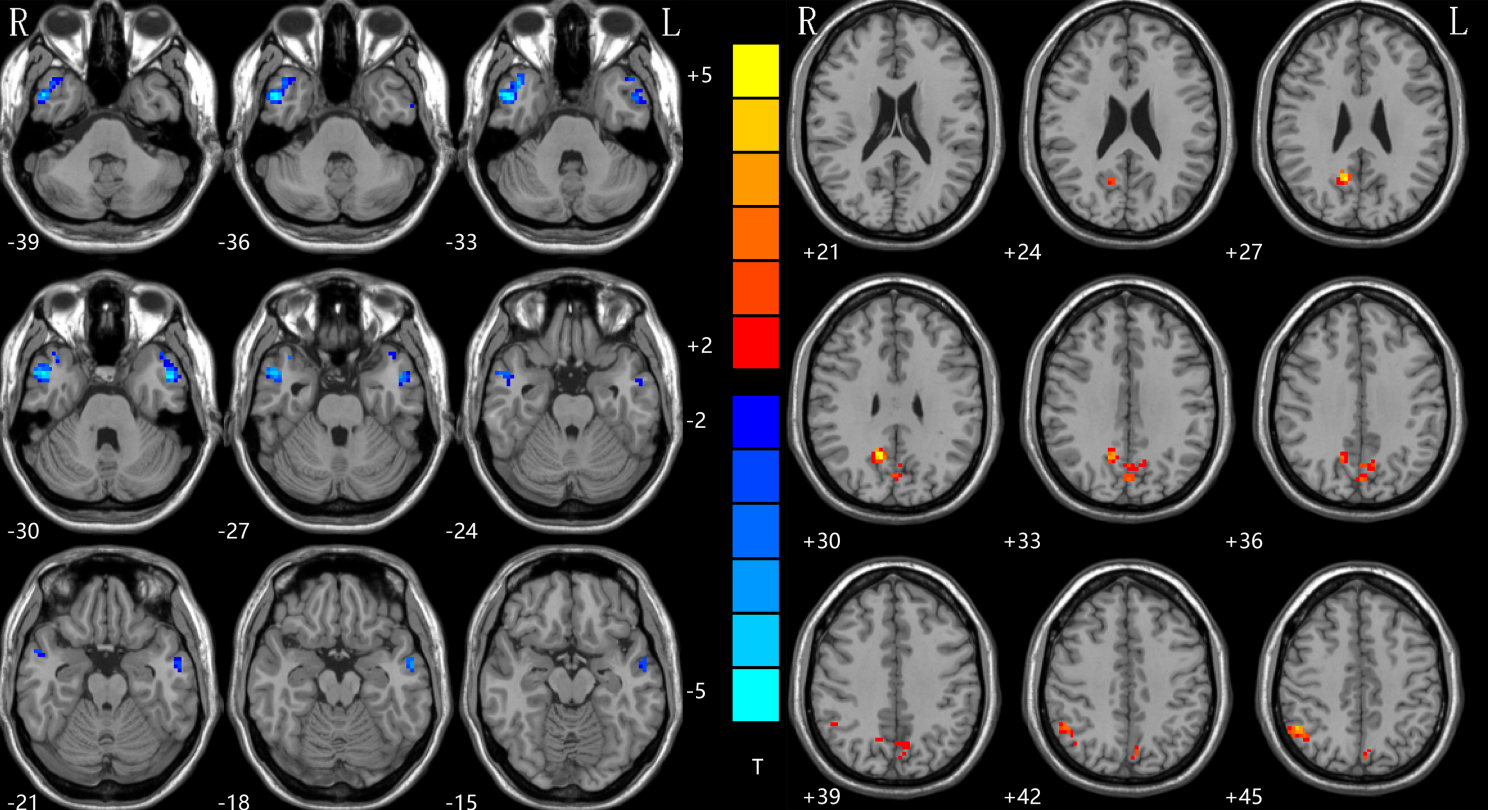
NH differences between patients with rTLE and HCs. Red and blue denote higher and lower NH, respectively, and the color bars represent the T values from the two-sample *t*-test of the group analysis. NH, network homogeneity; rTLE, right temporal lobe epilepsy; HCs, healthy controls.

With the two-sample *t-*tests *via* voxel-wise, cross-subject comparisons, significant differences were observed within the DMN, between the NH values for the patient and control groups. Compared to HCs, the rTLE patients had decreased NH in the right inferior temporal gyrus (ITG) and left middle temporal lobe (MTG), but increased NH in the bilateral precuneus (PCu) and right inferior parietal lobe (IPL) (Table 2, Figure 1).

**Table 2:** Signification differences in NH values between the groups.

**Cluster location**	**Peak X**	**(MNI) Y**	**Z**	**Number of voxels**	**T value**
Patients < controls					
Right ITG	51	6	−36	135	−4.80
Left MTG	−48	−3	30	70	−4.64
Patients >controls					
Right PCu	12	−60	30	45	4.59
Left PCu	0	−78	33	50	3.59
Right IPL	51	−57	48	47	4.16

*MNI, Montreal Neurological Institute; ITG, inferior temporal gyrus; MTG, middle temporal lobe; PCu, Precuneus; IPL, inferior parietal lobe*.

### Correlations Between NH and Clinical Variables

The mean NH values were extracted in the four regions (right ITG, left MTG, bilateral PCu, and right IPL), with significant group differences. Pearson's linear correlation analyses were performed between NH and these clinical variables in the patient group: RT; illness duration; and age at seizure onset. Results showed no significant correlation between NH and these clinical variables.

### SVM Results

**Figure 2:**
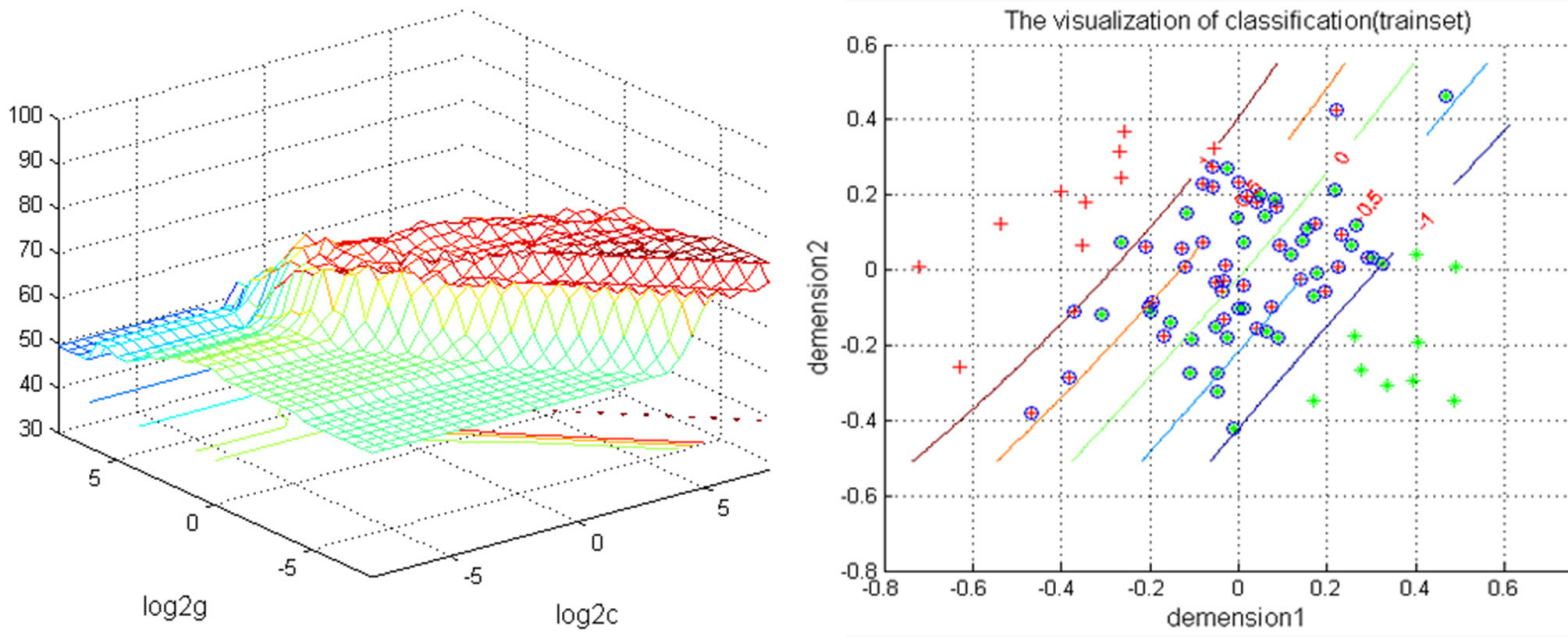
Depiction of classifications based on the SVM using a combination of NH values in the bilateral PCu to differentiate rTLE patients from HCs. Left: SVM parameters result of 3D view. g means gamma, c means penalty coefficient. Right: dimension 1 and dimension 2 represent the NH values in the bilateral PCu. Green crosses represent rTLE patients, and the red crosses represent HCs. SVM, support vector machine; NH, network homogeneity; PCu, precuneus; rTLE, right temporal lobe epilepsy; HCs, healthy controls.

The increased NH in the bilateral PCu in the rTLE patients were analyzed by the SVM method with a classification accuracy of 74.12%, a sensitivity of 72.01%, and a specificity of 72.81% (Figure 2).

## Discussion

NH is a new approach for detecting specific loci of compromised connectivity, and for studying within-network coherence. It has been used to study several diseases, such as attention deficit/hyperactivity disorder (39), major depressive disorder (54, 55), schizophrenia (42) and mild cognitive impairment (56). We applied this method to estimate the DMN homogeneity in TLE at rest. The results showed that rTLE patients have a decreased NH in the right ITG and left MTG, but increased NH in the bilateral PCu and right IPL when compared with HCs.

In TLE, it is widely considered that the temporal lobe plays an important part in the regulation and propagation of epileptic discharges, because of the presentation of epileptic foci. Hence, the temporal lobe has proven to be a common target for both structural and functional study of TLE. One study showed altered intrinsic functional connectivity in the temporal regions during both the latent and chronic periods of TLE (4). Among the numerous studies, aberrant regional activation of ITG and/or MTG were repeatedly found from neuroimaging. The ITG, with the localization of lateral and inferior surface of the temporal neocortex, is thought to be the central region for language formulation, and a tertiary visual association cortex region (57), which related to cognitive functions such as memory, language, and visual perception (58, 59). Consistent results from neuroimaging studies of major depressive disorder have demonstrated that this region is involved in emotional processing and social cognition (46, 60). Moreover, the ITG is a key node in a widespread network of frontal, temporal, parietal, occipital, and sub-cortical structures. Thus, abnormal activation of this region could significantly impair the function of the temporal lobe. The MTG plays a critical role in semantic memory and language processing (61). As a result, abnormal activation in the MTG could also consequently affect the function of the temporal lobe. In this study, we demonstrated decreased NH in the right ITG and the MTG. Accordingly, these abnormalities could impair memory and language functions, and result in dysfunction in rTLE patients.

The PCu, a key region of the DMN, is selectively connected with the intraparietal sulcus, the inferior and superior parietal lobules, and the caudal parietal operculum. Acting in concert, these structures are involved in the processing of visuo-spatial information (62–64). It is a significant and integrative structure which exhibits widespread connectivity with some cortical and sub-cortical regions (65). The PCu is responsible for various, essential, cognitive and behavioral functions, including episodic memory retrieval, visuospatial imagery, self-processing operations, and consciousness (65, 66). The right IPL is crucial in the DMN and the frontal parietal network, participating in sustaining attention, alertness, and task switching. Studies in rTLE using resting state fMRI, found that the right IPL had lower functional connectivity (FC) in TLE when compared with the control group (14, 67), which might result in alertness impairment in patients. Because the parietal lobe is connected to the temporal lobe, epileptic discharges from the epileptogenic zone (right temporal lobe) can spread to distant brain regions through the superior longitudinal fasciculus. However, we did not find the same activation pattern in our study. The discrepancy in findings might be attributed to there different epileptic focal positions in patients recruited in previous studies. A smaller sample size, or the application of different methods might also have influenced the results. Here, the inconsistent result may relate to the different analysis methods. Another probable explanation is as follows: according the roles of IPL and PCu in the cognition process, the increased NH in the bilateral PCu and right IPL might be a compensatory function for the damage to the temporal lobe, and this function becomes stronger depending on the severity of the temporal lobe damage. In addition, by measuring the abnormal NH values of lTLE, our previous study suggested that NH could be utilized as a neuroimaging biomarker for monitoring lTLE progression (unpublished). In this study, SVM analysis showed that increased NH values in the bilateral PCu could be used to distinguish rTLE patients from HCs with an accuracy of 74.12% (63/85), a sensitivity 72.01% (31/43), and a specificity 72.81% (31/42).

In the network mode of the human brain, DMN is characterized with a group of brain regions that are functionally consistent, that is, high activity while in a resting state, but decreased activity during non-specific task execution such as paying attention. It is closely related to the mental activities of other advanced cognitive functions, such as introspection, scene memory, environmental monitoring and awareness levels (68–72). A previous study confirmed that the DMN changed, which may be relevant to altered cognition and memory in TLE (13, 14, 73). Consistent with the studies referred to above, we thought that the DMN is dysfunctional in TLE, thereby negatively influencing memory and cognition in TLE patients. Furthermore, our study showed a dissociation pattern of activity in DMN, with hypoactivity in anterior regions of the DMN (right ITL and left MTL), but hyperactivity in posterior regions of the DMN (bilateral PCu and right IPL). Other studies of TLE have found significant differences in activity in the resting-state of the DMN, which may explain the symptoms of patients with TLE, such as loss of consciousness, impairments to learning and memory, emotions; and motor, sensory, or psychiatric symptoms (74, 75). The consistent results indicated that the DMN was disturbed, and this aberrance plays an important role in the pathophysiology of TLE.

It is worth noting that patients with rTLE had longer RTs, but no significant correlations between abnormal NH values and RT were found. As studies have demonstrated, TLE patients usually exhibit executive functional impairment. Since it is universally acknowledged that the DMN plays a crucial role in executive functions, we speculate that the regions showing abnormal NH in this study indirectly participate in executive functions. No significant correlations were found between abnormal NH values and RT, nor age of seizure onset or illness duration. These observations might imply that the abnormal NH values for the DMN, might be a trait change in rTLE patients.

There are several limitations to this study. First, the patients were not drug naïve, which might influence the results. Second, we could not thoroughly remove the physiological noise at rest, such as cardiac and respiratory rhythms using a 2-s repetition time, and that may bias the results. Third, this study focused on the DMN. Understanding the neurophysiological abnormalities of the DMN in rTLE would be helpful. For the same reason, some meaningful findings from other brain regions besides this network may have been excluded. Lastly, previous studies have shown that there are some morphological differences between the Chinese population and the others (76). For this reason, the use of the Chinese brain atlas for the data processing in this study may also partly limit the results.

In conclusion, the altered NH in the right ITG, left MTG, left MTG and bilateral PCu may be state-related changes of rTLE. And, the increased NH in the bilateral PCu may be a potential neuroimaging biomarker for rTLE.

## Data Availability Statement

The original contributions presented in the study are included in the article/supplementary material, further inquiries can be directed to the corresponding authors.

## Ethics Statement

The studies involving human participants were reviewed and approved by the Ethics Committee of the Taihe Hospital, Hubei University of Medicine. The patients/participants provided their written informed consent to participate in this study.

## Author Contributions

All authors listed have made a substantial, direct, and intellectual contribution to the work and approved it for publication.

## Funding

This work was funded by Health Commission of Hubei Province Scientific Research Project: (Grant Nos. WJ2019H232 and WJ2019H233).

## Conflict of Interest

The authors declare that the research was conducted in the absence of any commercial or financial relationships that could be construed as a potential conflict of interest. The reviewer LL declared a shared affiliation with the author YiZ at the time of review.

## Publisher's Note

All claims expressed in this article are solely those of the authors and do not necessarily represent those of their affiliated organizations, or those of the publisher, the editors and the reviewers. Any product that may be evaluated in this article, or claim that may be made by its manufacturer, is not guaranteed or endorsed by the publisher.
